# Enhanced Functional Recovery from Spinal Cord Injury in Aged Mice after Stem Cell Transplantation through HGF Induction

**DOI:** 10.1016/j.stemcr.2017.01.013

**Published:** 2017-02-16

**Authors:** Morito Takano, Soya Kawabata, Shinsuke Shibata, Akimasa Yasuda, Satoshi Nori, Osahiko Tsuji, Narihito Nagoshi, Akio Iwanami, Hayao Ebise, Keisuke Horiuchi, Hideyuki Okano, Masaya Nakamura

**Affiliations:** 1 Department of Orthopaedic Surgery, Keio University School of Medicine, 35 Shinanomachi, Shinjuku-ku, Tokyo 160-8582, Japan; 2 Department of Physiology, Keio University School of Medicine, 35 Shinanomachi, Shinjuku-ku, Tokyo 160-8582, Japan; 3 Genomic Science Laboratories, Dainippon Sumitomo Pharma Co., Ltd., 2-6-8 Doshoumachi, Chuo-ku, Osaka 541-0045, Japan

**Keywords:** aging, spinal cord injury, neural stem cell transplantation

## Abstract

The number of elderly patients with spinal cord injury (SCI) is increasing worldwide, representing a serious burden for both the affected patients and the community. Previous studies have demonstrated that neural stem cell (NSC) transplantation is an effective treatment for SCI in young animals. Here we show that NSC transplantation is as effective in aged mice as it is in young mice, even though aged mice exhibit more severe neurological deficits after SCI. NSCs grafted into aged mice exhibited better survival than those grafted into young mice. Furthermore, we show that the neurotrophic factor HGF plays a key role in the enhanced functional recovery after NSC transplantation observed in aged mice with SCI. The unexpected results of the present study suggest that NSC transplantation is a potential therapeutic modality for SCI, even in elderly patients.

## Highlights

• Aged mice exhibited more severe neurological deficits after SCI than young mice
• Grafted NSCs showed better survival in aged mice
• Aged mice exhibited enhanced functional recovery after NSC transplantation
• HGF was essential for efficient functional recovery in aged mice with SCI

## Graphical Abstract

**Figure:**
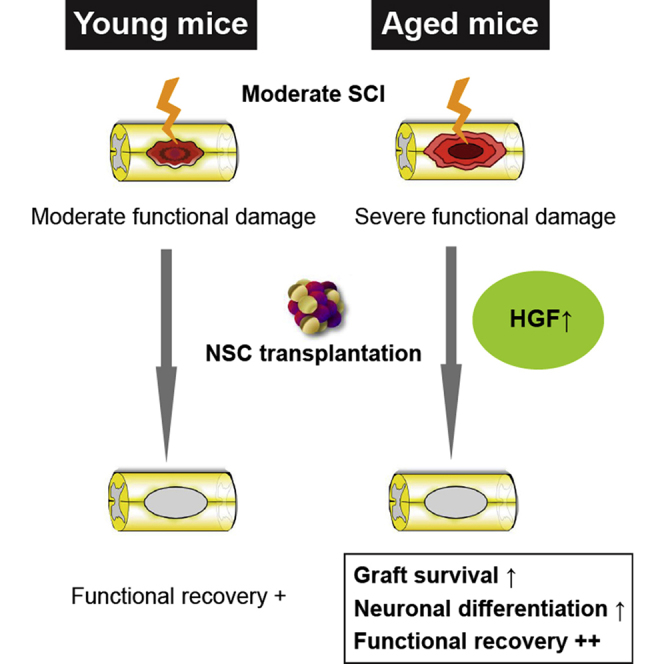

## Teaser

In this article, Nakamura, Okano, and colleagues report that NSC transplantation therapy is as effective in aged mice as it is in young mice, despite the more severe damage after SCI in aged mice. HGF is a key factor in the enhanced functional recovery after NSC transplantation observed in aged mice with SCI.

## Introduction

In the United States, approximately 12,000 patients are newly diagnosed with spinal cord injury (SCI) annually (Sahni and Kessler, 2010). Patients with SCI often develop permanent and devastating neurologic deficits and disabilities that may impose major burdens on themselves and society. Although approximately half of SCIs occur in adolescents and young adults between the ages of 16 and 30 years, the number of patients over 60 years with SCI has increased in recent years (Pickett et al., 2006, van den Berg et al., 2010). It is thus important to develop a better understanding of the pathophysiology of SCI in elderly patients and to provide improved therapeutic options for these patients.

Due to the poor regenerative capacity of the CNS, there are few treatment options for SCI, and these options yield only modest clinical benefits at best. However, emerging data suggest that cell transplantation (TP) therapies represent a potential therapeutic intervention for SCI. We and others have shown that cell TP into animals with SCI significantly improves neuronal defects and stimulates functional recovery (Barnabe-Heider and Frisen, 2008, Mothe and Tator, 2012, Nakamura and Okano, 2012). While the precise mechanisms underlying the beneficial effects of cell TP to the injured spinal cord are not fully understood, potential mechanisms include the replacement of lost cells, neuroprotection and trophic support, and the facilitation of axon outgrowth (Sahni and Kessler, 2010). Although the results of previous studies appear promising and may serve as a basis for future clinical applications, most of them have been performed using relatively young animal models. Thus, even though the number of elderly patients with SCI is currently on the rise, few studies have focused on the pathophysiology of SCI and the efficacy of cell TP therapy in aged animals. In general, the regenerative capacity of damaged tissues, including those of the CNS, declines with age, so even if cell TP therapy proves effective for younger patients with SCI, it may have only limited efficacy, or may not even be applicable, for aged patients.

To address these issues, we examined the efficacy of neural stem cell (NSC) TP in aged mice with SCI. Our results show that aged mice have the capacity to support the survival and differentiation of grafted cells and do so even more efficiently than younger mice. Furthermore, we identified hepatocyte growth factor (HGF) as a crucial factor in this enhanced functional recovery of aged mice. Taken together, our results shed light on the pathophysiology of SCI in aged animals and suggest that cell TP represents a potentially effective therapy for both young and elderly patients with SCI.

## Results

### Aged Mice Exhibit Distinct Gene Expression Patterns after SCI

**Figure 1:**
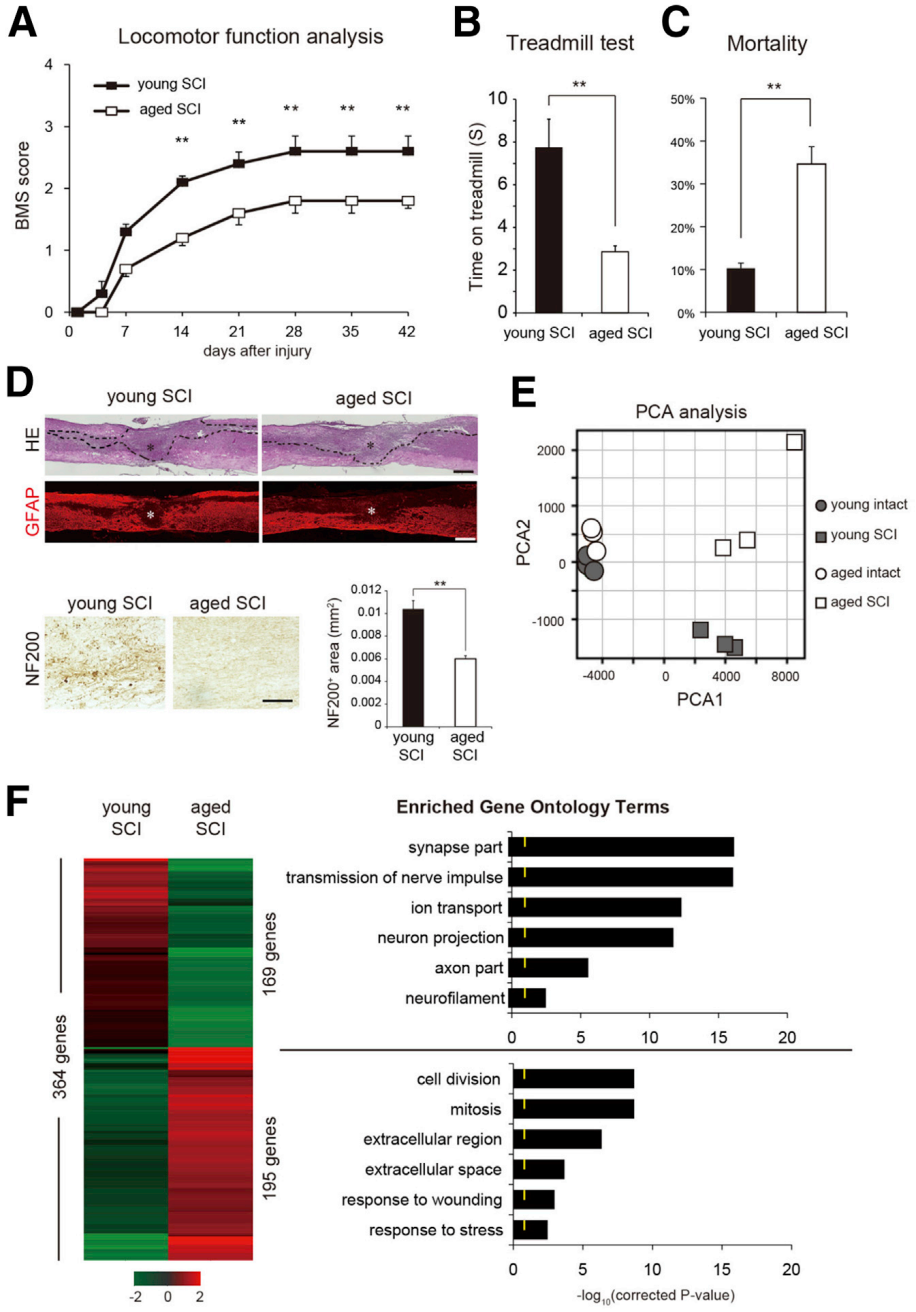
After SCI, Aged Mice Exhibit More Severe Defects than Young Mice (A) Time courses of changes in the BMS scores of the young and aged mice after SCI (n = 5 mice/group). ^∗∗^p < 0.01. (B) Rotarod treadmill test performed 6 weeks after SCI (n = 5 mice/group). ^∗∗^p < 0.01. (C) Mortality rates of the young and aged mice after SCI (n = 4 independent experiments, young SCI: 2/24, 1/9, 2/15, 1/12; aged SCI: 8/22, 2/10, 5/14, 4/11). ^∗∗^p < 0.01. (D) Representative images of H&E-stained and GFAP-immunostained sagittal sections of the spinal cord 9 days after SCI (upper panels: ^∗^indicates the lesion epicenter). The damaged areas are enclosed by dashed lines. Scale bars, 500 μm. Representative images of NF200-immunostained sections of the injured spinal cord and quantification of the NF200-positive cells in the aged and young mice (lower panels; n = 4 mice/group). Scale bar, 100 μm. ^∗∗^p < 0.01. (E) Principal component analysis (PCA) of the mRNA microarray data from the spinal cord samples collected from the young and aged mice with and without SCI. (F) Heatmap depicting the mRNA microarray profile of the spinal cord samples collected from the aged mice with SCI relative to that of the young mice with SCI (left panel). Up- and downregulated genes are shown in shades of magenta and green, respectively. Ontology analysis of the genes that were differentially expressed between the young and aged mice with SCI (right panel). Yellow marks indicate p = 0.5. Values are means with SEMs.

We first sought to better understand how aging affects SCI and the recovery from neurological deficits caused by SCI. Contusion SCIs were induced by an impactor at the Th9 level in young (2- to 3-month-old) and aged (15- to 18-month-old) mice (Table S1). In accordance with previous studies (Genovese et al., 2006, Siegenthaler et al., 2008), the aged mice exhibited less functional recovery from SCI than the young mice, as assessed by evaluations of locomotor function based on Basso Mouse Scale (BMS) scores (Basso et al., 2006), a locomotor rating scale based on the frequency analyses of seven locomotor categories, and rotarod treadmill tests (Figures 1A and 1B). Five weeks after SCI, 80% of the young mice were able to partially support their body weight on their back paws, whereas 90% of the aged mice could barely move their legs. Furthermore, the mortality rate after SCI was significantly higher in the aged than in the young mice (Figure 1C). Histological analyses of spinal tissue collected 9 days after SCI revealed that the volumes of the damaged areas was significantly larger in the aged mice than in the young mice (Figure 1D). In addition, there were few neurofilament 200 (NF200)-positive cells in the lesion epicenters in the aged than in the young mice.

To gain insight into the differences in the effects of SCI between young and aged mice at the molecular level, we next investigated the gene expression profiles of the spinal cords in both groups 9 days after SCI (sub-acute phase); this time period is thought to be the optimal time window for cell TP therapy (McDonald et al., 1999, Okada et al., 2005). Principal component analysis (PCA) revealed significant differences in the gene expression profiles between the young and aged mice (Figure 1E). In contrast, control animals without SCI exhibited similar expression patterns regardless of age, indicating that aging did not significantly affect the regulation of gene expression in the spinal cord. There were also no significant differences in spinal cord sizes or BMS scores between the young and aged mice under normal conditions, except for the rotarod test, on which the aged mice performed worse than the young ones at higher rotation speeds (Figures S1A–S1D). Global gene expression profile analyses of the injured spinal cords revealed significant differences in the expression levels of 364 genes between the aged and young mice. Among these genes, 169 were downregulated and 195 were upregulated in the aged compared with the young mice. The downregulated genes were mostly involved in the regulation of synapse-, ion transport-, or axon-related functions, whereas upregulated genes were implicated in regulation of the cell cycle, cell stress responses, or maintenance of the extracellular matrix (Figure 1F). Taken together, these observations indicated that the severity of SCI increases with age and that the gene expression profile of the spinal cord after injury, but not of the intact spinal cord, significantly differ between young and aged animals.

### Aged Mice with SCI Exhibit Enhanced Recovery after NSC TP

**Figure 2:**
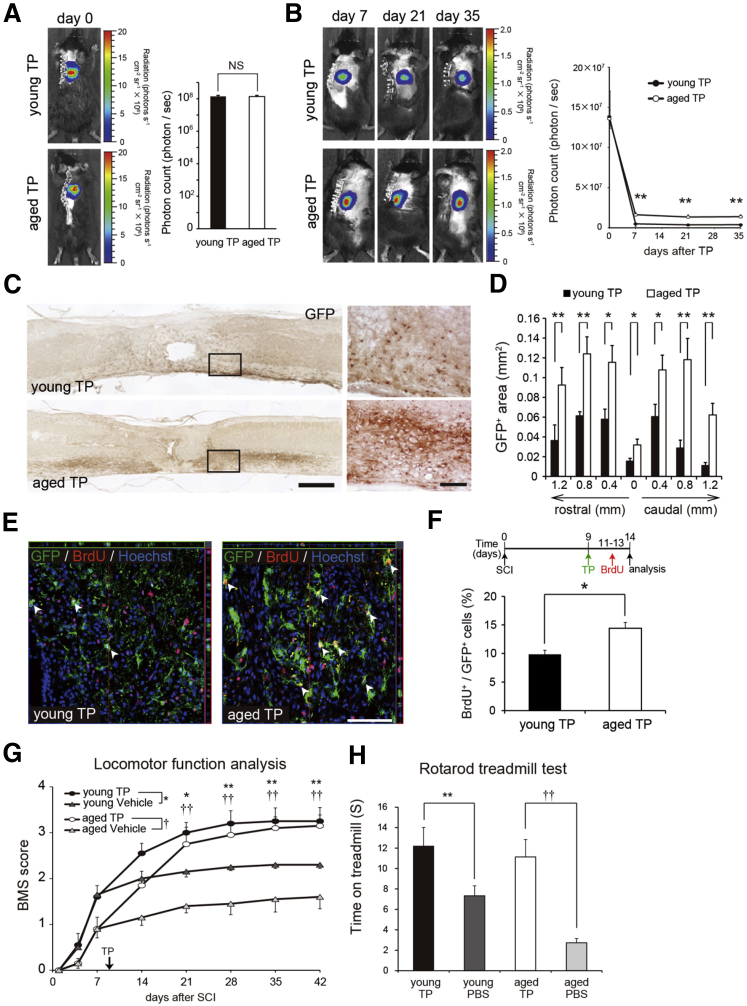
NSC TP Is as Effective in Aged Mice as It Is in Young Mice after SCI (A and B) Representative bioluminescence images and photon-count quantifications for young and aged mice after TP on days 0 (A), 7, 21, and 35 (B) (n = 10 mice/group). ^∗∗^p < 0.01. (C) Representative GFP-immunostained images of sagittally sectioned spinal cords collected 5 weeks after TP. Scale bars, 500 μm (left), 50 μm (right). (D) Axial sections of the spinal cord after TP were stained for GFP, and the GFP-positive area was quantified (n = 5 mice/group). ^∗^p < 0.05, ^∗∗^p < 0.01. (E) Representative images stained for GFP and bromodeoxyuridine (BrdU). The experimental plan is illustrated in (F), upper panel. Arrowheads indicate GFP^+^ BrdU^+^ cells. Scale bar, 50 μm. (F) Quantification of BrdU^+^ GFP^+^ NSCs from the experiments shown in (E) (n = 4 mice/group). ^∗^p < 0.05. (G) Time courses of the BMS scores of vehicle control mice (vehicle) and mice with NSC TP (n = 10 mice/group). ^∗^p < 0.05, ^∗∗^p < 0.01, ^††^p < 0.01. (H) Rotarod treadmill test performed 6 weeks after SCI (n = 10 mice/group). ^∗∗^p < 0.01, ^††^p < 0.01. Values are means with SEM.

Given that aging negatively affects the recovery from SCI, we next investigated whether the efficacy of NSC TP in aged mice with SCI differed from that in young mice. NSCs were prepared from the striata at embryonic day 14.5 of transgenic mice ubiquitously expressing fluorescent protein Venus-fused luciferase (CAG-*ff*Luc transgenic mice) (Hara-Miyauchi et al., 2012) and transplanted into the lesion epicenters of young and aged mice 9 days after SCI. The NSCs derived from the CAG-*ff*Luc transgenic mice expressed GFP and were capable of differentiating into neurons, astrocytes, and oligodendrocytes in vitro (Figures S2A and S2B) (Okada et al., 2005). A linear relationship between the number of living cells and the photon count by bioluminescence imaging (BLI) was confirmed (Figure S2C). We then examined the survival of the grafted NSCs via BLI at 7, 21, and 35 days after TP. Surprisingly, we found significantly higher photon counts in the aged than in the young mouse group (Figures 2A and 2B). Consistent with this observation, histological analyses revealed greater numbers of GFP^+^ cells in the aged than in the young mice after NSC TP (Figures 2C and 2D). Notably, we also found that bromodeoxyuridine incorporation into the grafted NSCs significantly increased in the aged compared with the young mice, indicating that the microenvironment in the aged mice is more suitable for the survival and proliferation of grafted NSCs than that in young mice (Figures 2E and 2F).

Behavioral analyses using the BMS and rotarod treadmill test revealed significant functional recovery in both the young and aged TP mouse groups compared with their respective vehicle control groups. Surprisingly, the aged TP mice group showed dramatic improvements in motor performance and BMS scores, comparable with those of the young TP mouse group. In contrast, the aged vehicle control group exhibited poorer motor performance than the young vehicle control group, as expected (Figures 2G and 2H). Moreover, NSC TP significantly improved the mortality rate in the aged mice after SCI to the same level seen in the young mice (young TP, 1/11; aged TP, 1/11). Histological evaluation indicated that NSC TP prevented atrophy of the spinal cord and demyelination in both the young and aged TP mice compared with their corresponding vehicle control mice (Figures S2D and S2E). Consistent with these observations, immunostaining for NF200 and 5-hydroxytryptamine also suggested that NSC TP enhanced axonal recovery in both the young and aged TP mouse groups (Figures S2F and S2G). Furthermore, we performed TP of adult skin fibroblasts (Fibro) derived from CAG-*ff*Luc transgenic mice into young and aged SCI mice as graft control. We found no significant difference in the survival rate of the grafted cells between young and aged Fibro TP mice (Figures S2H and S2I). TP of Fibro did not promote the functional recovery in either young or aged SCI mice and there were no significant differences in BMS scores between young and aged Fibro TP groups at 5 weeks after SCI and thereafter compared with their respective vehicle control groups (Figures S2J and S2K).

### Grafted NSCs Efficiently Differentiate into Neurons and Contribute to Polysynaptic Reconnection after SCI in Aged Mice

**Figure 3:**
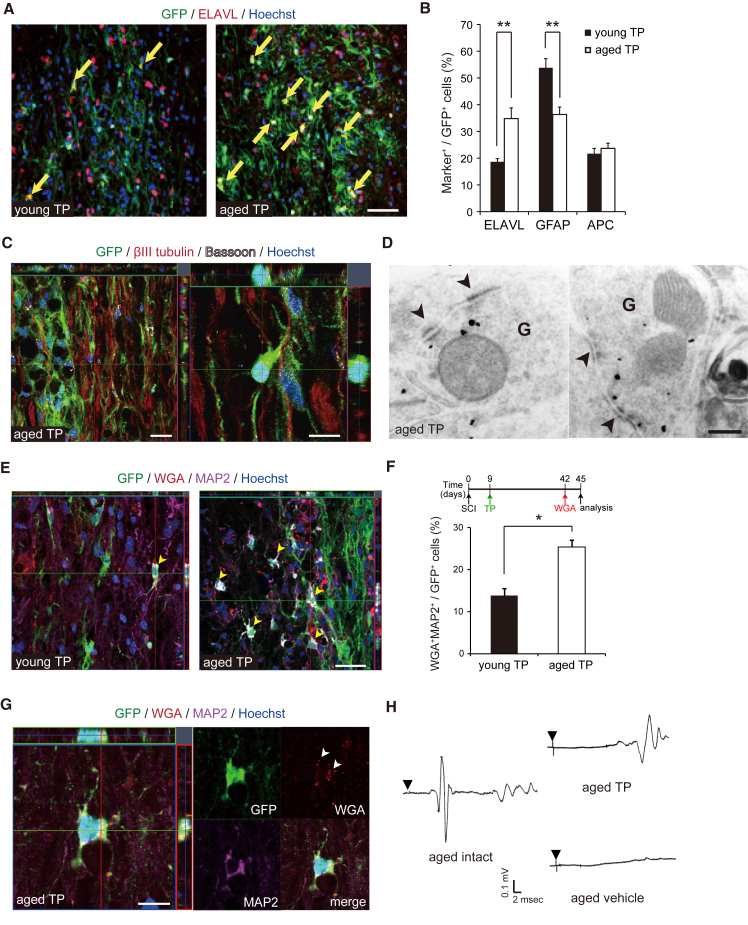
Neurons Derived from the Grafted NSCs Contribute to Functional Recovery in Aged Mice after SCI (A) Representative images of GFP^+^ ELAVL^+^ graft-derived neuronal cells (arrows) in the young and aged mice after TP. Scale bar, 50 μm. (B) Quantification of grafted NSC-derived neurons, astrocytes, and glial cells in the young and aged mice after TP (n = 5 mice/group). ^∗∗^p < 0.01. (C) Representative immunostained images of the synapses of aged mice after TP. Scale bars, 20 μm, 10 μm. (D) Electron-microscope images of synapses that formed between the host neurons and grafted GFP^+^ (black) neurons in aged mice after TP. G, grafted cells; arrowheads, synapse. Scale bar, 0.2 μm. (E) Representative images of WGA^+^ MAP2^+^ grafted NSC-derived cells (arrowheads) at the lesion epicenters of the young and aged mice with TP 3 days after WGA injection into the motor cortex. Scale bar, 50 μm. (F) Experimental plan (upper panel) and quantification of WGA^+^ MAP2^+^ NSC-derived cells in the experiments in (E) (n = 4 mice/group). ^∗^p < 0.05. (G) Representative magnified image of WGA^+^ MAP2^+^ NSC-derived cells (arrowheads) in an aged mouse after TP. Scale bar, 10 μm. (H) Electrophysiological transmission across the lesion site in aged mice.

To further explore the potential mechanisms of the enhanced recovery of the aged mice after cell TP, the fates of the grafted NSCs were examined histologically. There were more ELAVL^+^ (a marker for neurons) grafted cells and fewer GFAP^+^ (a marker for astrocytes) grafted cells in the aged mice than in the young mice after TP, indicating that the grafted NSCs tended to differentiate into a neuronal lineage rather than an astroglial one (Figures 3A and 3B). On the other hand, there was no significant difference in the frequency of APC^+^ (a marker for oligodendrocytes) grafted cells between the aged and young mice. Immunostaining for the presynaptic marker Bassoon and electron microscopic examination confirmed that the grafted NSC-derived neurons contributed to the formation of synapses in the aged mice after TP (Figures 3C and 3D). To determine whether the grafted NSC-derived neurons were integrated into the host neural circuitry, wheat germ agglutinin (WGA), a transsynaptically transported tracer (Kinoshita et al., 2002), was injected into the motor cortex 5 weeks after TP. Immunostaining revealed that there were more WGA^+^ MAP2^+^ (a marker for neurons) neurons among the GFP^＋^ graft-derived cells in the aged mice than in the young mice, suggesting that the grafted NSC-derived neurons formed connections with descending corticospinal fibers more efficiently in the aged mice than in the young mice (Figures 3E–3G). Furthermore, to evaluate the functional recovery after TP, motor-evoked potentials were measured 7 weeks after SCI (Figure 3H). In intact aged mice, stimulation at the C1 level evoked a short latency response (7.47 ± 0.43 ms), whereas this response was completely abolished in aged vehicle control mice with SCI. In contrast, the evoked responses were partially restored in three of four aged SCI mice after NSC TP (13.3 ± 0.92 ms), suggesting that the grafted cells contributed to polysynaptic reconnection. Similarly, the young intact mice showed a short latency response (7.62 ± 0.54 ms), which was completely abolished in the young vehicle control mice with SCI, and restored in the young SCI mice after NSC TP (8.05 ± 0.45 ms) (Figure S3). These observations together suggested that the grafted cells contributed to re-myelination and exhibited some trophic effects, as reported previously (Yasuda et al., 2011).

### HGF Is Induced in Aged Mice after SCI and Contributes to Their Enhanced Recovery

**Figure 4:**
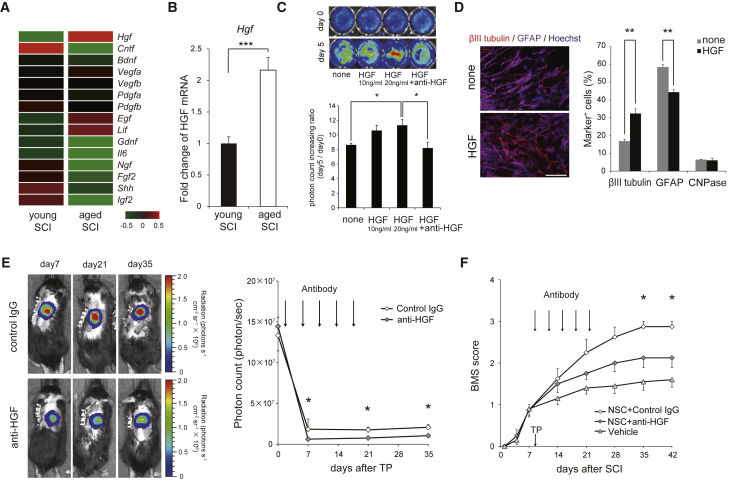
HGF Is Involved in Efficient Functional Recovery after SCI in Aged Mice (A) Comparative mRNA microarray analysis of the spinal cord samples of the young and aged mice collected 9 days after SCI. (B) Quantitative analysis of the expression levels of *Hgf* transcripts in the injured spinal cord (n = 6 mice/group). ^∗∗∗^p < 0.001. (C) Bioluminescence images and quantification of photon counts of the cultured NSCs derived from CAG-*ff*Luc transgenic mice. NSCs were cultured in the presence of HGF (400 ng/mL) for 5 days (n = 3 independent experiments). ^∗^p < 0.05. (D) NSCs incubated with or without HGF for 5 days were stained with antibodies against βIII-tubulin and GFAP (right panels). Quantification of βIII-tubulin^+^, GFAP^+^, and CNPase^+^ cells (n = 3 independent experiments). Scale bar, 50 μm. ^∗∗^p < 0.01. (E and F) Administration of an HGF-neutralizing antibody to the aged mice hindered the survival of grafted NSCs (E) and the recovery of motor function after SCI (F) (n = 4 mice/group). ^∗^p < 0.05. Values are means with SEM.

The enhanced survival and differentiation capacities of the grafted NSCs in the aged mice suggested neurotrophic factor(s) were induced upon SCI in these mice. Comparison of the microarray data gathered from the aged and young mice 9 days after SCI (Figure 1F) revealed significant differences in the expression levels of several neurotrophic factors. Among these factors, *Hgf* (which encodes HGF) was the most highly induced in the aged mice (Figures 4A and 4B). Immunohistochemical analyses revealed positive staining for HGF in the GFAP^+^ astrocytes and CD11b^+^ microglia in the aged mice following SCI (Figure S4A). Because HGF has been shown to promote functional recovery after SCI (Kitamura et al., 2007, Kitamura et al., 2011), we further explored the potential contributions of this factor in the aged mice with SCI. As reported previously (Kokuzawa et al., 2003), we found that HGF promoted NSC proliferation and neuronal differentiation in vitro (Figures 4C and 4D). Furthermore, the functional inhibition of HGF with neutralizing antibodies significantly reduced the survival and neuronal differentiation of the grafted NSCs (Figures 4E and S4B–S4D). The functional inhibition of HGF was confirmed by the decreased phosphorylation of the HGF receptor MET (Figures S4E and S4F). Consistent with these findings, treatment with HGF-neutralizing antibodies significantly reduced the functional recovery after SCI in the aged mice (Figure 4F). To investigate the potential role of HGF in the recovery of transsynaptic neuronal pathways following SCI, we performed anterograde tracing by injecting WGA into the cerebral cortex 5 weeks after TP. The results revealed that there were significantly fewer WGA^+^ MAP2^+^ grafted cells in the HGF-neutralizing antibody-treated mice than in the control immunoglobulin G (IgG)-treated mice, indicating that HGF enhances synapse formation between the grafted NSC-derived neurons and the descending corticospinal fibers (Figures S4G and S4H). In addition, spinal cord atrophy and demyelination were more severe in the HGF-neutralizing antibody-treated mice than in the control IgG-treated mice (Figures S4I and S4J). Taken together, these findings suggest that the enhanced recovery from SCI of the aged mice was at least partly dependent on the production of HGF in the microenvironment of the injured spinal tissue.

## Discussion

Aging negatively affects the regenerative capacity of tissues and organs. This holds true for the spinal cord, and aged animals and patients exhibit more severe neuronal defects, poorer functional recovery, and higher mortality rates than their younger counterparts following SCI (Furlan and Fehlings, 2009, Genovese et al., 2006, Gwak et al., 2004, Scivoletto et al., 2003). However, the present study showed that the aged animals exhibited enhanced functional recovery after NSC TP, suggesting that the outcomes of cell TP therapy do not necessarily reflect the regenerative capacity.

Aged animals exhibit increased inflammatory reactions, increased pro-inflammatory cytokine production, and reduced remyelination after SCI (Genovese et al., 2006, Kumamaru et al., 2012, Siegenthaler et al., 2008). In addition, the number of endogenous stem cells that can potentially contribute to neuronal regeneration in the adult CNS is decreased (Maslov et al., 2004). These age-related adverse effects are thought to be causally related to the impaired functional recovery of aged animals after SCI. Consistent with this potential relationship, PCA of the mRNA microarray data in the present study showed significant differences in the gene expression profiles of the spinal tissues of aged and young mice following SCI, which may account for the poorer recovery of the aged mice after SCI. However, we speculated that these same differences also underlie the enhanced functional recovery of the aged mice after NSC TP. It is also worth mentioning that there are some discrepancies between our findings and those of previous studies, which showed that fetal cells grafted in the brain of aged animals functioned more poorly than did those grafted in young mice (Collier and Sortwell, 1999, Shetty et al., 2008). Although the reasons for the discrepancy remain to be elucidated, it may be due to differences in the microenvironment between the spinal cord and brain or to differences in the biological properties of the grafted cells used in each study.

Our comparative microarray analyses revealed increases in several inflammatory cytokines (Kumamaru et al., 2012) and several neurotrophic factors in the aged mice after SCI. Among these cytokines and neurotrophic factors, we identified host-derived HGF as a critical regulator in the enhanced survival and differentiation of the grafted NSCs. HGF is a potent mitogen and a crucial neurotrophic factor in the CNS. Notably we previously identified HGF as a potent agent for the promotion of functional recovery in primates and mice after SCI (Kitamura et al., 2007, Kitamura et al., 2011). The results of these studies and the present one underscore an important role of HGF in spinal regeneration.

The results of the transcriptome and behavioral analyses of the present study also indicate that the spinal cord may not suffer from cellular senescence, and indeed may retain most of its functions, at least to the age of 18 months in normal mice. In contrast, the spinal cord appears to manifest a senescent phenotype upon injury. Interestingly, recent studies have suggested that senescent cells undergo changes in protein production and secretion that ultimately lead to a state called the senescent-associated secretory phenotype (SASP) (Coppe et al., 2010, Tchkonia et al., 2013). Senescent cells that have acquired this phenotype produce various secreted proteins and generate a microenvironment that promotes the survival and proliferation of tumor cells. By analogy, it is tempting to speculate that a SASP-like phenotype emerges in the spinal cord upon injury and that this condition renders the injury's microenvironment in aged mice more suitable for the grafted cells. It is important to note, however, that the production of cytokines (a feature of the SASP-like phenotype) in the injured spinal cord is transient, subsiding within a few weeks (Kumamaru et al., 2012), and therefore does not result in the chronic inflammation associated with SASP. Nevertheless, it is important to understand the mechanisms that underlie the distinct gene expression patterns of aged mice after SCI, because this information might contribute to improvements in the survival and growth of grafted cells in both old and young subjects.

Our study has some limitations. To examine the effects of the non-neural control graft, we performed TP of adult skin Fibro derived from the same immunophenotype as the NSCs. These data showed that aged Fibro TP mice showed less recovery than did young Fibro TP mice, and that there was no significant difference in the survival rate of the grafted cells between young and aged Fibro TP mice. However, to do this experiment in detail, all groups (NSCs, PBS, Fibro) must be evaluated simultaneously considering the time interval between experiments and the variability of SCI. Therefore, an appropriate evaluation of the non-neural cell TP for aged SCI should be examined in future studies.

In conclusion, our data show that aged mice with SCI exhibit enhanced regenerative capability following NSC TP, even though they suffer more severely from the SCI than do young mice. Our results also indicated that the injured spinal cord of aged mice confers a permissive environment for the survival and growth of the grafted cells via an increased production of HGF. Although these findings should be interpreted with caution due to the differences between mouse models and human patients, they may have important clinical implications in the establishment of cell-based therapeutic modalities for patients with SCI.

## Experimental Procedures

### Mice

All of the experiments were performed with female C57BL/6J mice. The mice were housed in groups under a 12-hr light/dark cycle with ad libitum access to food and water. All experiments were performed in accordance with the Guidelines for the Care and Use of Laboratory Animals of Keio University School of Medicine.

### SCI Model

Young and aged mice were anesthetized via intraperitoneal injections of ketamine (100 mg/kg) and xylazine (10 mg/kg). After laminectomy at the Th9 spinal vertebra, the dorsal surface of the dura mater was exposed, and SCI was induced using a commercially available SCI device (a 70-kdyn impact was delivered with an Infinite Horizon Impactor [Precision Systems & Instrumentation]). The motor function of the hind limbs after SCI was evaluated using the locomotor rating test of the BMS (Basso et al., 2006) and the rotarod treadmill test (Ogura et al., 2001) (Muromachi Kikai).

### NSC Culture and TP

NSCs were cultured and expanded as described previously (Reynolds and Weiss, 1992). In brief, the striata of CAG-*ff*Luc transgenic mice (Hara-Miyauchi et al., 2012) on embryonic day 14 were dissociated using a fire-polished glass pipette. The dissociated cells were collected by centrifugation and resuspended in culture medium followed by cell-cluster (neurosphere) formation. For differentiation, the neurospheres were cultured without serum for 5 days. Nine days after injury, 5 × 10^5^ NSCs were transplanted into the lesion epicenters of the young and aged mice using a glass micropipette and a stereotaxic injector (KDS310; Muromachi Kikai). An equal volume of PBS was injected into the control mice.

### Statistical Analyses

All values are presented as the mean ± SEM. One-way ANOVA followed by the Tukey-Kramer test for multiple comparisons was used to determine the significance of the differences in the histological quantifications, rotarod treadmill tests, and MEP experiments. Repeated-measures two-way ANOVA, followed by the Tukey-Kramer test, was used for the BMS and BLI analyses. Significance was defined as p < 0.05 in all statistical analyses. GraphPad Prism software (version 5.0d) was used for the analyses (GraphPad).

## Author Contributions

M.T., S.K., S.S., A.Y., S.N., O.T., and H.E. performed experimental work, data analysis, and reviewed the manuscript. M.T., N.N., A.I., K.H., H.O., and M.N. were responsible for the experimental design, data analysis, and review of the manuscript.

## Accession Numbers

The DNA microarray data have been submitted to the NCBI under accession number GEO: GSE93561.

## Supplemental Information

Document S1. Supplemental Experimental Procedures, Figures S1–S4, and Table S1

Document S2. Article plus Supplemental Information
