# Supplementary material for: Enhanced Functional Recovery from Spinal Cord Injury in Aged Mice after Stem Cell Transplantation through HGF Induction
Source: Stem Cell Reports. 2017 Feb 16;8(3):509–18. doi: 10.1016/j.stemcr.2017.01.013 (PMC5355635; doi:10.1016/j.stemcr.2017.01.013)
Supplement: Document S1. Supplemental Experimental Procedures, Figures S1–S4, and Table S1 [file mmc1.pdf]

Stem Cell Reports, Volume *8*

Supplemental Information

Enhanced Functional Recovery from Spinal Cord Injury in Aged Mice after Stem Cell Transplantation through HGF Induction

Morito Takano, Soya Kawabata, Shinsuke Shibata, Akimasa Yasuda, Satoshi Nori, Osahiko Tsuji, Narihito Nagoshi, Akio Iwanami, Hayao Ebise, Keisuke Horiuchi, Hideyuki Okano, and Masaya Nakamura

Figure S1

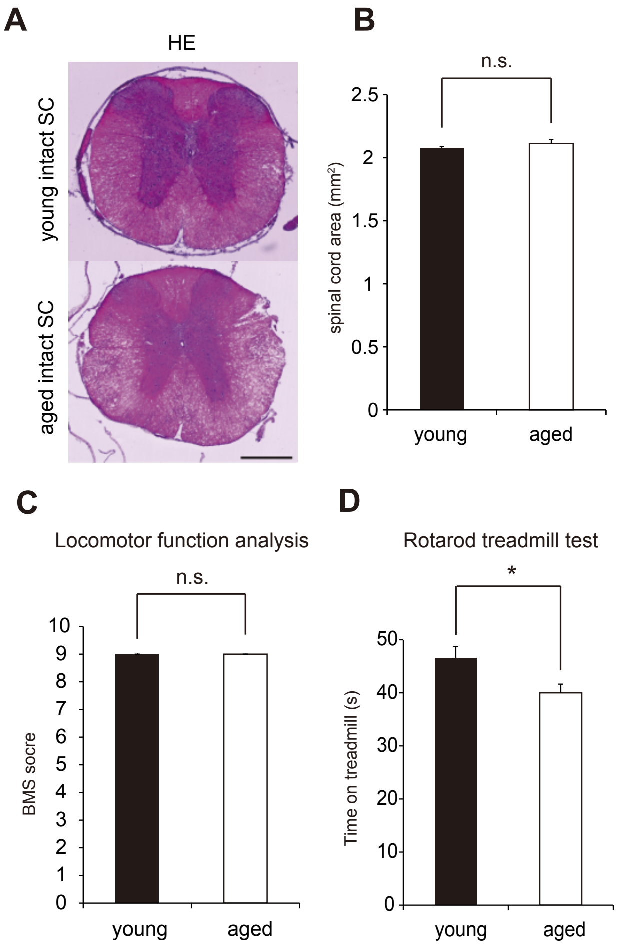

A
HE
young intact SC
aged intact SC
B
n.s.
spinal cord area (mm²)
2.5
2
1.5
1
0.5
0
young
aged
C
Locomotor function analysis
n.s.
BMS socre
10
9
8
7
6
5
4
3
2
1
0
young
aged
D
Rotarod treadmill test
*
Time on treadmill (s)
50
40
30
20
10
0
young
aged

Figure S2

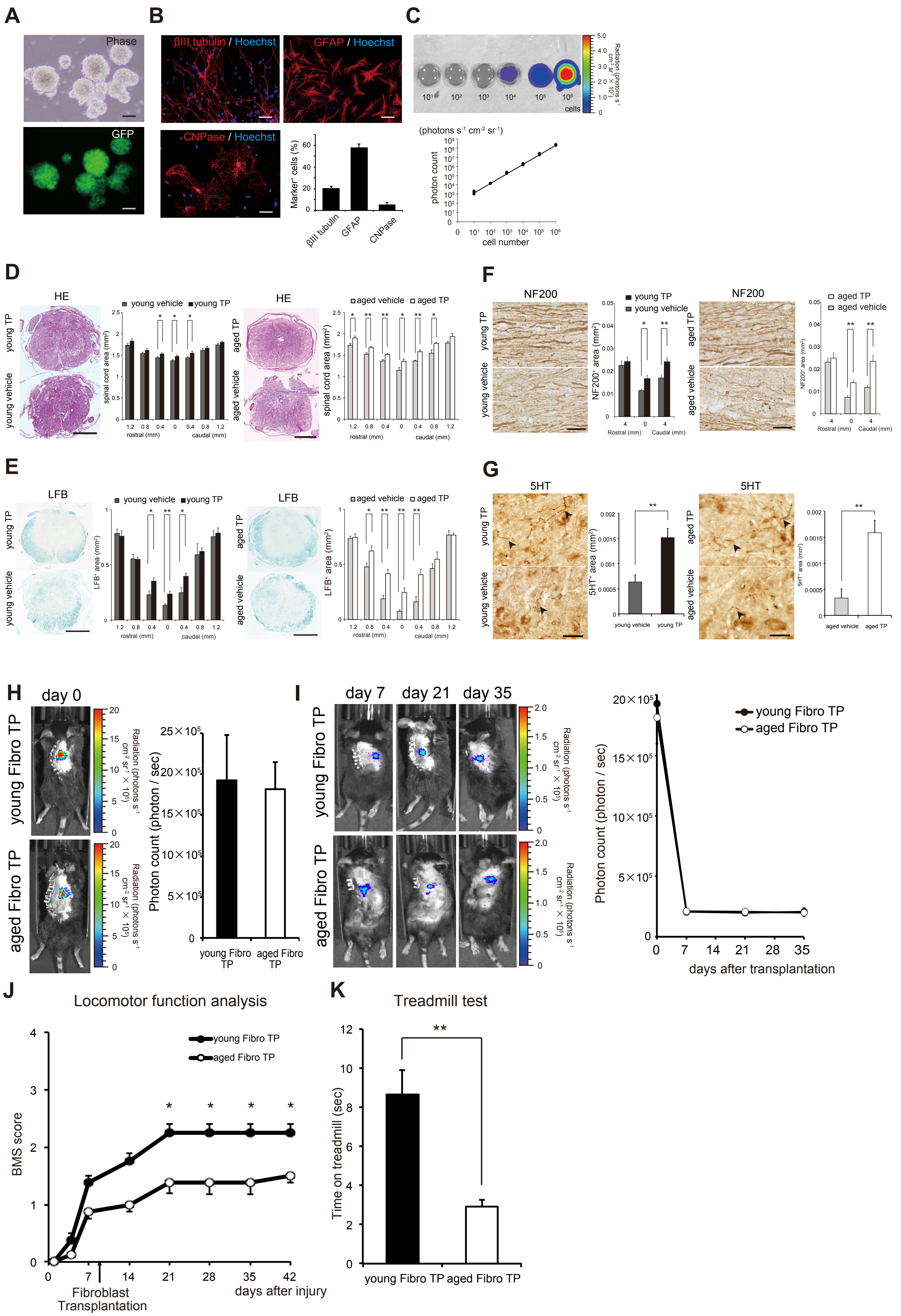

A
Phase
GFP
B
βIII tubulin / Hoechst
GFAP / Hoechst
CNPase / Hoechst
Marker+ cells (%)
βIII tubulin
GFAP
CNPase
C
cells
(photons s-1 cm-2 sr-1)
photon count
cell number
D
HE
young TP
young vehicle
young vehicle
young TP
spinal cord area (mm2)
rostral (mm)
caudal (mm)
aged TP
aged vehicle
aged vehicle
aged TP
E
LFB
LFB+ area (mm2)
F
NF200
NF200+ area (mm2)
Rostral (mm)
Caudal (mm)
G
5HT
5HT+ area (mm2)
H
day 0
young Fibro TP
aged Fibro TP
Photon count (photon / sec)
I
day 7
day 21
day 35
days after transplantation
J
Locomotor function analysis
BMS score
Fibroblast Transplantation
days after injury
K
Treadmill test
Time on treadmill (sec)
young Fibro TP
aged Fibro TP

Figure S3

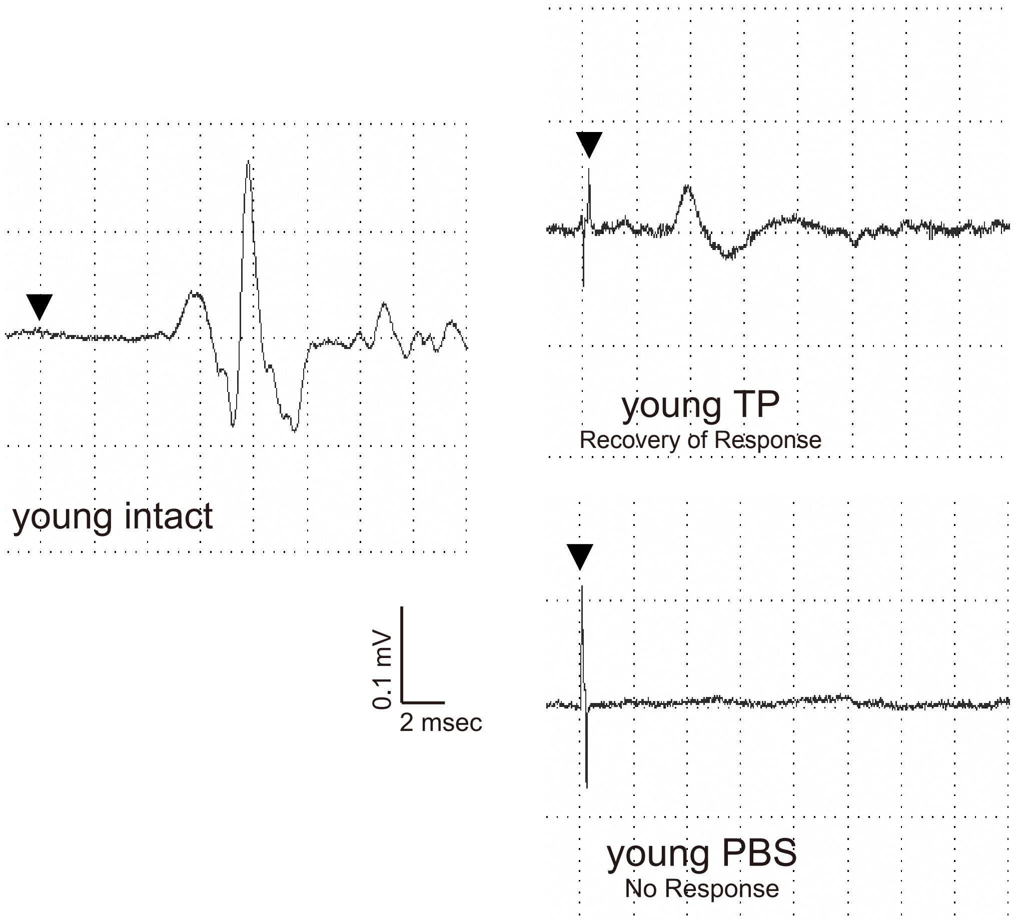
young intact
young TP
Recovery of Response
young PBS
No Response
0.1 mV
2 msec

Figure S4

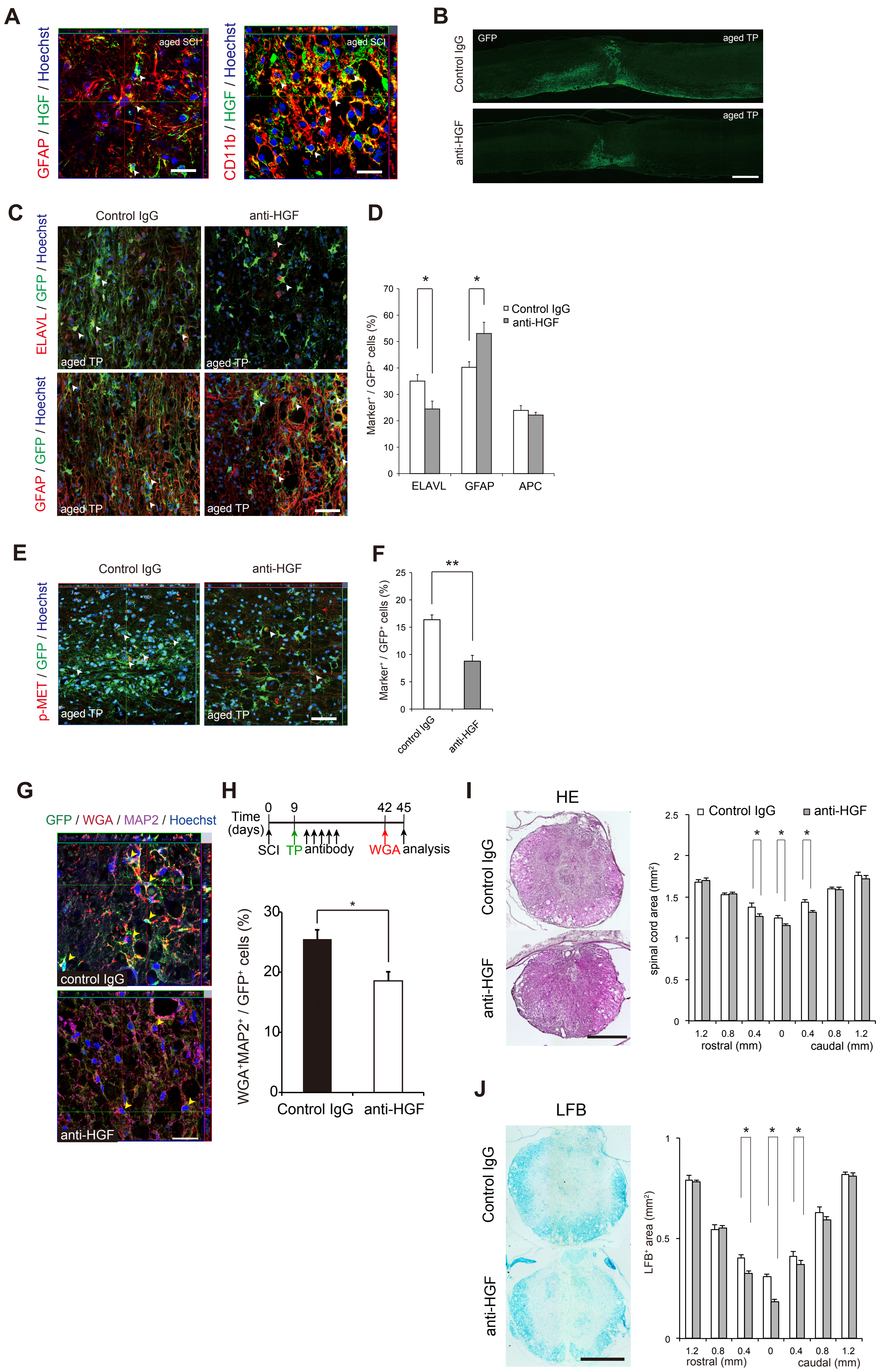
A
GFAP / HGF / Hoechst
aged SCI
CD11b / HGF / Hoechst
aged SCI
B
Control IgG
GFP
aged TP
anti-HGF
aged TP
C
Control IgG
anti-HGF
ELAVL / GFP / Hoechst
GFAP / GFP / Hoechst
aged TP
D
Control IgG
anti-HGF
Marker⁺ / GFP⁺ cells (%)
ELAVL
GFAP
APC
E
Control IgG
anti-HGF
p-MET / GFP / Hoechst
aged TP
F
Marker⁺ / GFP⁺ cells (%)
control IgG
anti-HGF
G
GFP / WGA / MAP2 / Hoechst
control IgG
anti-HGF
H
Time (days)
0 9 42 45
SCI TP antibody WGA analysis
WGA⁺MAP2⁺ / GFP⁺ cells (%)
Control IgG
anti-HGF
I
HE
Control IgG
anti-HGF
spinal cord area (mm²)
rostral (mm)
caudal (mm)
J
LFB
Control IgG
anti-HGF
LFB⁺ area (mm²)
rostral (mm)
caudal (mm)

Table S1. Summary of mice used in this study

	Exp1	Exp2	Exp3	Exp4	Exp5	Exp6	Total
Young mice group							
normal	5	0	0	0	0	0	5
SCI	24(2)	9(1)	15(2)	12(1)	0	4	64(6)
Histology 9 d (SCI)	4	0	0	0	0	0	4
RNA 9 d (SCI)	6	0	0	0	0	0	6
Transplantation (NSC)	0	5(1)	6	8	0	0	19(1)
Histology 35 d (TP)	0	4	6	0	0	0	10
MEP 35 d (TP&PBS)	0	0	8	0	0	0	8
BrdU 11-13 d (TP)	0	0	0	4	0	0	4
WGA 42 d (TP)	0	0	0	4	0	0	4
Transplantation (PBS)	0	4	6	0	0	0	10
Transplantation (Fibro)	0	0	0	0	0	4	4
Aged mice group							
normal	5	0	0	0	0	0	5
SCI	22(8)	10(2)	14(5)	11(4)	38(7)	26(6)	121(32)
Histology 9 d	4	0	0	0	0	0	4
RNA 9 d	6	0	0	0	0	0	6
Transplantation (NSC)	0	6(1)	5	4	14(1)	9(1)	38(3)
Histology 35 d (TP)	0	5	5	0	0	0	10
MEP 35 d (TP&PBS)	0	0	8	0	0	0	8
BrdU 11-13 d (TP)	0	0	0	0	4	0	4
WGA 42 d (TP)	0	0	0	4	0	0	4
control IgG (TP)	0	0	0	0	4	0	4
anti-HGF (TP)	0	0	0	0	5(1)	0	5(1)
Transplantation (PBS)	0	3(1)	9(5)	3(1)	7(1)	0	22(8)
Transplantation (Fibro)	0	0	0	0	0	6(2)	6(2)
Control IgG WGA (TP)	0	0	0	0	0	4	4
anti-HGF WGA (TP)	0	0	0	0	0	4	4

Number of mice used (unplanned deaths)

Supplemental Figure Legends

Figure S1. Comparison of axial sections of the spinal cord at the Th9 level and motor performance of young and aged mice under normal conditions (related to Figure 1)

(A) HE-stained axial sections of intact spinal cords obtained from young and aged mice. Scale bar: 500 μm.

(B) Quantitative analysis of HE-stained areas of spinal cord axial sections (n=4 mice/group). n.s., not significant.

(C) BMS scores of young and aged mice (n=5 mice/group). n.s., not significant.

(D) Quantification of the rotarod treadmill test (n=5 mice/group). *p<0.05. Values are means with SEMs.

Figure S2. Histological and bioluminescence evaluations of the spinal cords of young and aged mice after NSC and Fibro TP (related to Figure 2)

(A) Fluorescence and phase-contrast images of NSCs derived from the E14.5 fetal brains of CAG-ffLuc transgenic mice. Scale bar: 50 μm.

(B) Representative images of differentiation-induced NSCs stained with anti-βIII tubulin (a maker for neurons), anti-GFAP (a marker for astrocytes), or anti-CNPase (a marker for oligodendrocytes) and quantification of the cells in each lineage. Scale bar: 50 μm.

(C) In vitro bioluminescence imaging and quantification of the photon count of CAG-ffLuc NSCs (n=3 independent experiments, R2=0.9924). Values are means with SEMs.

(D) Representative images of HE-stained axial spinal cord sections of young and aged mice with SCI treated with NSC TP or with PBS (vehicle) and quantitative analysis of the spinal cord areas in HE-stained axial sections from different regions (n=5 mice/group). Scale bar: 500 μm. *p<0.05, **p<0.01.

(E) Representative images of LFB-stained axial spinal cord sections of young and aged mice with SCI treated with NSC TP or PBS (vehicle) and quantitative analysis of the myelinated areas revealed in LFB-stained axial sections from different regions (n=5 mice/group). Scale bar: 500 μm. *p<0.05, **p<0.01.

(F) Representative images of sagittal sections stained for NF200 at lesion epicenters of young and aged mice with SCI treated with NSC TP or with PBS (vehicle) and quantitative analysis of NF200-positive areas (n=5 mice/group). Scale bar: 500 μm. *p<0.05, **p<0.01.

(G) Representative images of axial sections stained for 5-hydroxytryptamine (5HT) at the lumbar intumescences of the young and aged mice with SCI treated with NSC TP or PBS and quantitative

analysis of the 5HT-positive areas (n=5 mice/group). Scale bar: 20 μm. **p<0.01. Values are means with SEM.

(H and I) Representative bioluminescence images and photon-count quantifications for young and aged mice after ffluc-fibroblast TP on days 0 (A), 7, 21, and 35 (B) (n=4 mice/group).

(J) Time courses of changes in BMS scores of young and aged SCI mice with fibroblast (n=4 mice/group). *p<0.05.

(K) Rotarod treadmill test performed 6 weeks after SCI (n=4 mice/group). *p<0.05. Values are means with SEMs.

Figure S3. Electrophysiological transmission across the lesion site in young mice (related to Figure 3)

The intact young mice showed a short latency response (7.62 ± 0.54 ms), which was completely abolished in young vehicle control mice with SCI. The evoked responses were well restored in young SCI mice after NSC TP (8.05 ± 0.45 ms).

Figure S4. Functional blockage with an anti-HGF antibody hindered the therapeutic effect of NSC transplantation in the aged TP group (related to Figure 4)

(A) Representative images of GFAP+ HGF+ cells (arrowheads) and CD11b+ HGF+ cells (arrowheads) in aged mice 9 days after SCI. Scale bar: 20 μm.

(B) Representative images of sagittal sections of the spinal cord stained for GFP. Scale bar: 500 μm.

(C) Representative images of sections stained for GFP/ELAVL and GFP/GFAP. Tissue samples were collected from aged mice treated with TP and either control IgG or anti-HGF antibody. Scale bar: 100 μm.

(D) Quantification of the immunostained cells in the experiments in (C) (n=4 mice/group). *p<0.05. Values are means with SEMs.

(E) Representative images of the sections stained for GFP/p-MET. Tissue samples were collected from the aged SCI mice treated with control IgG or anti-HGF antibody. Scale bar: 100 μm.

(F) Quantification of p-MET-positive cells in the experiment in (E) (n=4 mice per group). **p<0.01. Values are means with SEMs.

(G) Representative images of WGA+ MAP2+ NSC-derived cells (arrowheads) at lesion epicenters of aged mice with SCI treated with TP and either control IgG or anti-HGF antibody, 3 days after WGA injection into the motor cortex. Scale bar: 50 μm.

(H) Experimental plan (upper panel) and quantification of WGA+ MAP2+ NSCs-derived cells in the experiments in (A) (n=4 mice/group). *$p<0.05$.

(I) Representative images of HE-stained axial spinal cord sections of the aged mice with SCI treated with TP and either control IgG or anti-HGF antibody, and quantitative analysis of the spinal cord areas in HE-stained axial sections from different regions (n=4 mice/group). Scale bar: 500 µm. *$p<0.05$.

(J) Representative images of LFB-stained axial spinal cord sections of aged mice with SCI treated with TP and administered with control IgG or anti-HGF antibody, and quantitative analysis of the myelinated areas revealed in the LFB-stained axial sections from different regions (n=4 mice/group). Scale bar: 500 µm. *$p<0.05$.

Supplemental Experimental Procedures

Fibroblast culture

Adult skin fibroblasts were obtained from 8-week-old CAG-fluorescent protein-fused Luciferase (*ff*Luc) transgenic mice (Hara-Miyauchi et al., 2012). To obtain these cells, skin was peeled from the body of adult mice, minced into 5-mm pieces, placed on culture dishes, and incubated in Dulbecco's modified Eagle's medium, containing 10% fetal bovine serum, 50 U penicillin, and 50 mg/ml streptomycin (Matsui et al., 2012). Cells that migrated out of the skin pieces were trypsinized and transferred to new plates. We used the adult mouse fibroblasts at passage 3-5 for fibroblast transplantation (Tsuji et al., 2011).

Bioluminescence imaging

A Xenogen-IVIS spectrum cooled charge-coupled device optical macroscopic imaging system (Caliper Life-Sciences, Hopkinton, MA, USA) was used for bioimaging. The NSCs were injected into the injured spinal cord with D-Luciferin (150 ng/ml), and the photon counts were examined on day 0. Thereafter, the survival of the NSCs was monitored for 5 weeks via intraperitoneal injections of D-Luciferin (0.3 mg/g body weight) and measurements of the photon counts as previously described (Okada et al., 2005).

Gene expression analyses

Injured and naive mice were anesthetized and transcardially perfused with heparinized saline (5 U/ml) 9 days after injury (n=3 each). Dissected segments of the spinal cord at the Th9 level were rapidly frozen and placed in TRIzol (Invitrogen). Total RNA was isolated using an RNeasy Mini Kit (Qiagen Inc., Hilgen, Germany), following the manufacturer's instructions. For microarray analyses, RNA quality was assessed using a 2100 Bioanalyzer (Agilent Technologies Inc., Santa Clara, CA, USA), and 100 ng of total RNA was reverse transcribed, biotin labeled, and hybridized to a GeneChip® Mouse Genome 430 2.0 Array (Affymetrix Inc., Santa Clara, CA, USA). The array was subsequently washed and stained in a Fluidics Station 450 according to the manufacturer's instructions. Microarrays were scanned using a GeneChip Scanner 3000 7G, and the raw image files were converted to normalized signal intensity values using the MAS 5.0 algorithm. PCA was performed using the Gene Spring GX software (Agilent Technologies Inc.). For cluster analyses, the normalized data were narrowed down using cutoff values for each expression signal (>50) and fold changes (i.e., the difference between the signal from the young injured spinal cords and that of the aged injured spinal cords was greater than 2). The heat map was created with GeneSpring GX. GO term enrichment was performed for the genes that exceeded the

above-described cut-off value (Mistry et al., 2012). qRT-PCR was performed using ABI 7900HT (Applied Biosystems) and TaqMan probes (Applied Biosystems).

Histological analyses

The mice were anesthetized and transcardially perfused with 4% paraformaldehyde in 0.1 M PBS. The spinal cords were removed, embedded in OCT compound (Sakura Finetech Co., Ltd., Tokyo, Japan), and sectioned in the sagittal or axial planes at 12 µm on a cryostat (Leica CM3050 S, Wetzlar, Germany). Spinal cords were histologically evaluated with hematoxylin-eosin (HE) staining, Luxol Fast Blue (LFB), and immunohistochemistry. Tissue sections were stained with the following primary antibodies: anti-GFAP (rat IgG, Invitrogen, CA, USA A11007), anti-GFP (rabbit IgG, Frontier Institute, Hokkaido, Japan Af2020), anti-BrdU (rat IgG, Abcam, Cambridge, UK ab6326), anti-ELAVL (Hu) (human IgG, a gift from Dr. Robert Darnell, Rockefeller University, NY, USA), anti-HGF (goat IgG, R&D Systems, Inc., MN, USA AF2207), anti-CD11b (rat IgG, Abcam, Cambridge, UK ab8878), anti-APC (mouse IgG, Abcam, Cambridge, UK ab16894), anti-βIII tubulin (mouse IgG, Sigma-Aldrich, MO, USA T8660), anti-Bassoon (mouse IgG2a, Stressgen, Brussels, Belgium SAP7F407), anti-MAP2 (mouse IgG1, Sigma-Aldrich M4403), anti-CNPase (mouse IgG1, Sigma-Aldrich C5922), anti-NF200 (mouse IgG1, Millipore, MA, USA MAB5262), anti-5HT (goat IgG, ImmunoStar, Hudson, WI, USA 20080), and anti-p-MET (goat IgG, Santa Cruz Biotechnology, CA, USA sc514148). For DAB staining, a biotinylated secondary antibody (Jackson ImmunoResearch Laboratory, Inc., PA, USA 200-002-211) was used after exposing the sections to 0.3% H2O2 for 30 minutes at room temperature to inactivate the endogenous peroxidase. Signals were enhanced with the Vectastain ABC kit (Vector Laboratories, Inc., CA, USA). Quantitative analyses of the histological findings were performed using a BZ 9000 microscope and Dynamic Cell Count BZ-HIC software (Keyence Co., Osaka, Japan). Threshold values were maintained at a constant level for all analyses. The GFP^{+}, HE-stained, and LFB-stained areas were quantified using images of the axial sections of the lesion epicenter and 0.4 mm, 0.8 mm, and 1.2 mm rostral and caudal to the epicenter at 100Å~ magnification (n=5 each). To quantify the NF200 fibers, four regions were automatically captured within the midsagittal sections of the lesion epicenter and 4.0 mm rostral and caudal to the epicenter at 400Å~ magnification (n=5 each). To assess the 5HT+ fibers, five automatically captured regions within the axial sections were analyzed at the lumbar intumescence (n=5 each). To quantify the proportions of each cell phenotype among the grafted cells *in vivo*, five regions were captured within sagittal sections of the lesion epicenter at 200Å~ magnification using an LSM 700 confocal laser-scanning microscope (Carl Zeiss, Munich, Germany). GFP^{+} and phenotypic marker double- or triple-positive cells were counted in each section (n=5 each).

Immuno-electron microscopy

Spinal cords collected from aged mice with TP were cut into 12-μm cryosections, which were then incubated with 5% Block Ace (DS Pharma Biomedical) and 0.1% saponin in 0.1 M phosphate buffer for 1 h. The sections were then immunostained with a primary rabbit anti-GFP antibody (1:100 MBL 598) for 72 h and a nanogold-conjugated anti-rabbit secondary antibody (1:100 Invitrogen N24916) for 24 h at 4°C. After 2.5% glutaraldehyde fixation, the nanogold signals were enhanced with an HQ-Silver kit (Nanoprobes Inc.) for 10 min. Samples were post-fixed with 0.5% osmium tetroxide; dehydrated with ethanol, acetone, and QY1 (N-butyl glycidyl ether); and embedded in Epon. Ultrathin (80 nm) sagittal spinal cord sections were stained with uranyl acetate and lead citrate for 10 and 12 min, respectively. The sections were examined under a transmission electron microscope (JEOL model 1230) and photographed using a Digital Micrograph 3.3 (Gatan Inc., CA, USA).

Electrophysiology

Electrophysiological experiments were conducted with an electromyography (EMG)/evoked potential measuring system (Neuropack S1 MEB-9400 series, Nihon Kohden, Tokyo, Japan). Young and aged SCI mice treated with TP or PBS were anesthetized with an i.p. injection of ketamine (40 mg/kg) and xylazine (4 mg/kg) as previously described (Nori et al., 2011). An electrode was inserted into the spinal cord in the occipito-cervical area to induce motor-evoked potential (MEPs). The potentials were recorded with two needle electrodes in each hindlimb. The ground electrode was placed subcutaneously between the coil and the recording electrodes. To induce MEPs, a 0.4-mA stimulus was applied at the electrode; the pulse duration in all experiments was 0.2 ms. The onset latency was measured as the time in milliseconds between the stimulus and the onset of the first wave. Ten responses were averaged and sorted for off-line analyses (Yasuda et al., 2011).

WGA tracing

To visualize selective and functional trans-synaptic neuronal pathways, WGA recombinant protein (10 mg/ml, 2 μl per cortex; Invitrogen, CA, USA) was injected into the motor cortex. In this system, WGA injected into well-mapped neural pathways labels polysynaptic neurons. WGA protein is efficiently transported through the axons and dendrites of host and grafted neurons.

Antibody administration

For in vitro neurosphere culture and differentiation assays, recombinant mouse HGF (10 and 20 ng/ml; R&D Systems, Inc., MN, USA) and an anti-HGF antibody (400 ng/ml; R&D Systems AF2207) were used. For in vivo functional blocking, a goat IgG-neutralizing antibody against mouse HGF (50 µg/kg; R&D Systems AF2207) and an isotype-matched control goat IgG (R&D Systems AB108C) were administered systemically via five intraperitoneal injections administered twice per week for two and one-half weeks beginning immediately after NSC transplantation in the aged mice**.**

Supplemental References

Hara-Miyauchi, C., Tsuji, O., Hanyu, A., Okada, S., Yasuda, A., Fukano, T., Akazawa, C., Nakamura, M., Imamura, T., Matsuzaki, Y., et al. (2012). Bioluminescent system for dynamic imaging of cell and animal behavior. Biochem Biophys Res Commun *419*, 188-193**.**

Tsuji, O., Miura, K., Okada, Y., Fujiyoshi, K., Mukaino, M., Nagoshi, N., Kitamura, K., Kumagai, G., Nishino, M., Tomisato, S., et al. (2010). Therapeutic potential of appropriately evaluated safe-induced pluripotent stem cells for spinal cord injury. Proc Natl Acad Sci U S A 107, 12704-12709

Matsui, T., Takano, M., Yoshida, K., Ono, S., Fujisaki, C., Matsuzaki, Y., Toyama, Y., Nakamura, M., Okano, H., Akamatsu, W. (2012). Neural Stem Cells Directly Differentiated from Partially Reprogrammed Fibroblasts Rapidly Acquire Gliogenic Competency. Stem Cells 30, 1109-1119

Mistry, D.S., Chen, Y.F., and Sen, G.L. (2012). Progenitor Function in Self-Renewing Human Epidermis Is Maintained by the Exosome. Cell Stem Cell 11, 127-135.

Nori, S., Okada, Y., Yasuda, A., Tsuji, O., Takahashi, Y., Kobayashi, Y., Fujiyoshi, K., Koike, M., Uchiyama, Y., Ikeda, E., et al. (2011). Grafted human-induced pluripotent stem-cell-derived neurospheres promote motor functional recovery after spinal cord injury in mice. Proc Natl Acad Sci U S A 108, 16825-16830.

Okada, S., Ishii, K., Yamane, J., Iwanami, A., Ikegami, T., Katoh, H., Iwamoto, Y., Nakamura, M., Miyoshi, H., Okano, H.J., et al. (2005). In vivo imaging of engrafted neural stem cells: its application in evaluating the optimal timing of transplantation for spinal cord injury. FASEB J 19, 1839-1841.

Yasuda, A., Tsuji, O., Shibata, S., Nori, S., Takano, M., Kobayashi, Y., Takahashi, Y., Fujiyoshi, K., Hara, C.M., Miyawaki, A., et al. (2011). Significance of remyelination by neural stem/progenitor cells transplanted into the injured spinal cord. Stem Cells 29, 1983-1994.
